# Exploring the neurobiology of the premonitory phase of migraine preclinically – a role for hypothalamic kappa opioid receptors?

**DOI:** 10.1186/s10194-022-01497-7

**Published:** 2022-09-30

**Authors:** Caroline M. Kopruszinski, Robson Vizin, Moe Watanabe, Ashley L. Martinez, Luiz Henrique Moreira de Souza, David W. Dodick, Frank Porreca, Edita Navratilova

**Affiliations:** 1 Department of Pharmacology, College of Medicine, University of Arizona, Tucson, AZ USA; 2 Department of Neurology, Mayo Clinic, Phoenix, USA; 3 Department of Collaborative Research, Mayo Clinic, Scottsdale, USA

**Keywords:** Kappa opioid receptors (KOR), Hypothalamus, Arcuate nucleus, Premonitory phase, Premonitory symptoms, Migraine prevention

## Abstract

**Background:**

The migraine premonitory phase is characterized in part by increased thirst, urination and yawning. Imaging studies show that the hypothalamus is activated in the premonitory phase. Stress is a well know migraine initiation factor which was demonstrated to engage dynorphin/kappa opioid receptors (KOR) signaling in several brain regions, including the hypothalamus. This study proposes the exploration of the possible link between hypothalamic KOR and migraine premonitory symptoms in rodent models.

**Methods:**

Rats were treated systemically with the KOR agonist U-69,593 followed by yawning and urination monitoring. Apomorphine, a dopamine D1/2 agonist, was used as a positive control for yawning behaviors. Urination and water consumption following systemic administration of U-69,593 was also assessed. To examine if KOR activation specifically in the hypothalamus can promote premonitory symptoms, AAV8-hSyn-DIO-hM4Di (Gi-DREADD)-mCherry viral vector was microinjected into the right arcuate nucleus (ARC) of female and male KOR^CRE^ or KOR^WT^ mice. Four weeks after the injection, clozapine N-oxide (CNO) was administered systemically followed by the assessment of urination, water consumption and tactile sensory response.

**Results:**

Systemic administration of U-69,593 increased urination but did not produce yawning in rats. Systemic KOR agonist also increased urination in mice as well as water consumption. Cell specific Gi-DREADD activation (i.e., inhibition through Gi-coupled signaling) of KOR^CRE^ neurons in the ARC also increased water consumption and the total volume of urine in mice but did not affect tactile sensory responses.

**Conclusion:**

Our studies in rodents identified the KOR in a hypothalamic region as a mechanism that promotes behaviors consistent with clinically-observed premonitory symptoms of migraine, including increased thirst and urination but not yawning. Importantly, these behaviors occurred in the absence of pain responses, consistent with the emergence of the premonitory phase before the headache phase. Early intervention for preventive treatment even before the headache phase may be achievable by targeting the hypothalamic KOR.

**Supplementary Information:**

The online version contains supplementary material available at 10.1186/s10194-022-01497-7.

## Background

Migraine is a multiphasic neurological disorder that commonly includes a premonitory period, aura in some patients, headache, post-drome and interictal stages [1, 2]. These phases often, but not always, occur in a temporal sequence, with the premonitory phase preceding the headache phase by hours to days [3–5]. The mechanistic basis of non-painful symptoms of migraine is relatively poorly understood [3]. These symptoms, however, can be as disabling as the migraine-related headache attack [3]. While the prevalence of individuals experiencing migraine premonitory symptoms is not fully known, several studies report that such symptoms occur in approximately 80% of adult individuals with migraine [6–8]. As premonitory symptoms generally suggest impending headache pain attack, interventions at this stage are usually more effective in preventing the subsequent disabling headache phase of this disorder. Whether neural mechanisms underlying the premonitory phase are directly linked to activation of pain pathways and the development of the headache during a migraine attack is not known. Further mechanistic understanding of the premonitory phase is essential to unraveling the early neurobiology of migraine, opening avenues for implementing new therapeutic strategies.

Sleep disturbance, fatigue, mood changes, yawning, thirstiness, food cravings, polyuria, and photophobia are the most common clinically reported premonitory symptoms [1–3]. The distinct premonitory features reported by patients implicate involvement of multiple areas of the brain [1–3, 5, 9–12]. One key area likely to be associated with many of the observed clinical symptoms is the hypothalamus [1–3, 5, 10–13]. Imaging studies have revealed that the hypothalamus is activated prior to the pain phase of migraine [5, 14–16]. Numerous hypothalamic neurotransmitters have also been implicated in migraine neurobiology, including dopamine, somatostatin, vasopressin, PACAP, oxytocin and orexin [3, 11, 12]. The hypothalamus is a part of the descending pain modulatory pathway and sends neural projections to brain regions known to be associated with migraine, including the midbrain periaqueductal gray (PAG) [1, 3, 5, 9, 10, 13, 17]. Hypothalamic dopaminergic cells also project to the trigeminocervical complex where trigeminal afferent nociceptive signals may be modulated [3, 13]. However, whether these, or other hypothalamic mechanisms may be linked to some, or all, premonitory symptoms has not been sufficiently investigated.

Stress, or relief of stress, has been commonly linked to onset of migraine attacks [1, 18–21]. Preclinical studies have shown that the dynorphin/kappa opioid receptor (KOR) system is activated by stress [21–24]. The dynorphin/KOR pathway is widely distributed in multiple brain regions, including the hypothalamus, and its activation could play a role in modulation of homeostasis, pain, sleep, appetite and others [25, 26]. Stressful events can promote a lack of sleep, increased fatigue and anorexia, all of which have been reported during the premonitory phase of migraine [27, 28], suggesting a possible link to hypothalamic KOR signaling. As stress engages KOR-expressing neurons in the hypothalamus of mice, including the arcuate nucleus (ARC) [25, 29, 30], we tested the hypothesis that systemic KOR agonists or cell-specific Gi-DREADD activation of ARC KOR^CRE^ expressing neurons could promote premonitory symptoms in rodents. Specifically, we evaluated possible links between KOR activation and increased urination, water consumption and yawning behaviors, as well as possible effects on pain behaviors.

## Methods

### Animals

Studies were performed using 200–250 g female and male Sprague Dawley rats (Harlan Laboratories, Indianapolis, IN, USA) and 6-week-old female and male C57BL6/J mice (Jackson Laboratory, Sacramento, CA, USA). KOR^CRE^ female and male mice [31] were kindly provided by Dr. Sarah E. Ross (University of Pittsburgh, Pittsburgh, PA, USA) and were crossed with C57BL/6 J mice for more than six generations; heterozygous mice are designated as KOR^CRE^, wild type littermates as KOR^WT^. A total of 6 female rats, 6 male rats, 38 female mice and 32 male mice were used in this study. Animals were housed in a room on a 12/12-h light/dark cycle (7 am to 7 pm lights on), with controlled temperature and humidity and with free access to food and water in the University of Arizona animal facility. A total of 125 animals were used in these studies, with 8—13 animals per group for behavior evaluation. All experimental procedures were performed in accordance with the ARRIVE guidelines, the ethical guidelines of the International Association for the Study of Pain regulations on animal welfare, and the National Institutes of Health guidelines for the care and use of laboratory animals. The experimental procedures were approved by the Institutional Animal Care and Use Committee of the University of Arizona. Animals were randomly divided into control, and experimental groups, and experiments were blinded for the treatment and/or genotype.

### Drugs

U-69,593 (Abcam, Cambridge, UK) was diluted in a 5% (2-Hydroxypropyl)-β-cyclodextrin (Sigma, St. Louis, MO, USA) solution and administered subcutaneously (s.c.) at 0.56 and 30 mg/kg. R-( −)-Apomorphine hydrochloride hemihydrate (Sigma, St. Louis, MO, USA) was diluted in saline and administered s.c. at 0.05 mg/kg. AAV8-hSyn-DIO-hM4Di (Gi-DREADD)-mCherry viral vector (44,362-AAV8, Addgene, Watertown, MA, USA) and was stereotaxically injected at 100 nL (1 × 1010 vg/100 nL at 20 nL/min speed) into the right ARC. Clozapine-N-Oxide (CNO) (TOCRIS, Minneapolis, MN, USA) was injected intraperitoneally (i.p.) at 5 mg/kg. Controls received the respective vehicles at 10 mL/kg.

### Stereotaxic surgery for virus injection in mice

Mice were positioned in a stereotaxic head holder (KOPF Instruments, Tujunga, CA, USA) under inhalational anesthesia (2–5% isoflurane). An incision was made on the animal’s skin to localize the skull suture bregma. A surgical drill (KOPF Instruments, Tujunga, CA, USA) was used to produce a hole in the skull followed by glass pipette insertion into the right ARC of the hypothalamus according to the proper coordinates (AP -1.4 mm, ML + 1.2 mm, DV -5.8 mm with a 10° angle). The Gi-DREADD virus was injected slowly (20 nL/min), glass pipet removed and the hole on the skull was closed with bone wax with the skin sutured. Animals were carefully monitored for 5 days after surgery until complete recovery. Mice were kept for four weeks at the animal care facility before the testing to allow the virus expression.

### Yawning in rats

Yawning is a common behavior in rats and has been widely studied. Rats were placed in individual clear Plexiglass chambers for a one-hour acclimation. A video camera was placed in front of the chambers. Animals then received an s.c. injection of apomorphine, U-69,593 or control. Yawning behavior was recorded for 120 min after the treatment, and the number of yawning episodes during this interval was counted.

### Assessment of polyuria and water consumption in mice and rats

Four weeks after hypothalamic administration of the Gi-DREADD virus, mice were placed in individual clear Nalgene metabolic cages (PGC International, Palm Desert, CA, USA) for one-hour acclimation with free access to water. After acclimation, all animals were injected subcutaneously with 1 mL of 0.9% of sodium chloride to allow hydration, followed by a systemic injection of CNO (5 mg/kg, i.p.) and were immediately placed back into the metabolic cages. Naïve mice received a systemic injection of either U-69,593 or vehicle instead of CNO. The total volume of urine and water consumption were quantified at 120 min after treatment. Increased volume of urine was considered as an outcome measure of polyuria (increased urination).

Quantitative evaluation of polyuria in rats was performed by placing individual absorbable underpads below each clear Plexiglass chamber. Rats were acclimated in the chambers for a one-hour followed by an s.c. injection of apomorphine, U-69,593 or vehicle. The weight of the absorbable underpads was collected prior to and 120 min after the treatment. The amount of urine was approximated by calculating the difference between the weight of the individual underpads before and after the treatment. Significant increase in the amount of urine in test groups compared to vehicle treated rats was considered as a measure of polyuria (increased urination).

### Evaluation of cephalic and extracephalic allodynia

The periorbital (cephalic) and hindpaw (extracephalic) tactile sensory responses were evaluated before and after the hypothalamic Gi-DREADD virus injection and CNO administration. Mice were placed individually in suspended clear Plexiglas chambers with wire mesh floors for two hours to habituate before testing. Tactile response frequency was measured by the perpendicular application of von Frey filaments (Touch Test sensory evaluators; Stoelting, Wood Dale, IL, USA) applied to the periorbital region (0.4 g filament), at the center of the forehead, or the plantar surface of the hindpaw (1 g filament). The filaments were applied 10 times each, and the number of responses after the filament application were counted. Swiping the face, shaking the head, and/or turning away from the stimuli were considered positive periorbital responses. Sharp withdrawal, shaking and/or licking the paw were considered positive hindpaw responses. Frequency response (in percent) was calculated as (number of positive responses/10) * 100%.

Baseline responses for periorbital and hindpaw tactile stimulation with von Frey filaments were collected in male and female mice prior to Gi-DREADD administration into the right ARC. After 4 weeks, baseline periorbital and hindpaw sensory responses were measured and KOR^CRE^ and KOR^WT^ mice received a single systemic injection of CNO followed by sensory responses assessment again intermittently for 5 h.

### Statistical analysis

**Fig. 1:**
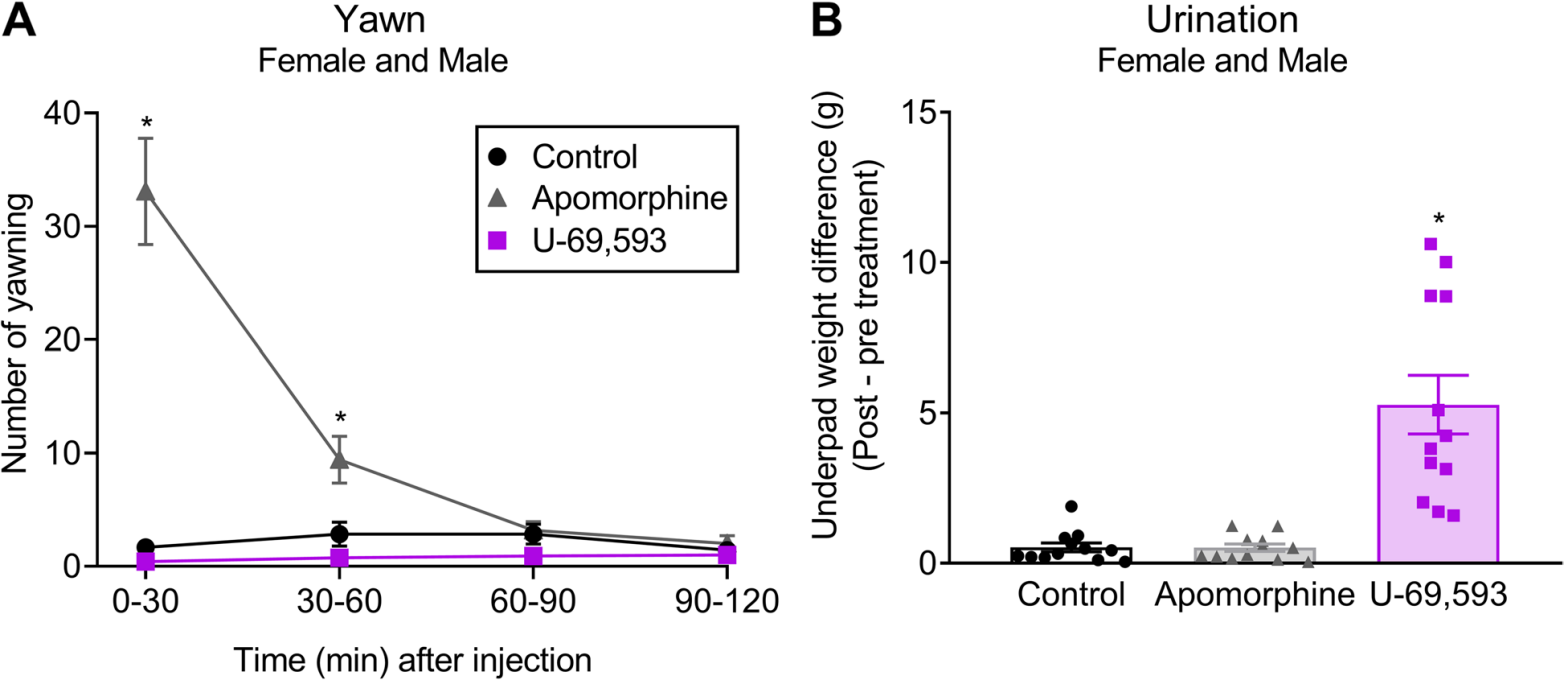
Systemic KOR agonist, U-69,593, failed to induce yawning behavior but increased urination in both female and male rats. **A** The number of yawning events and, **B** the increase in urination were evaluated for 120 min after a single subcutaneous administration of U-69,593 at 0.56 mg/kg, apomorphine at 0.05 mg/kg (a non-selective dopamine agonist used as a positive control for yawning) and vehicle-control. The number of yawning was quantified in 30-min intervals. Urination was quantified as the difference between the weight of the individual absorbable underpads before drug administration and 120 min after testing. Female and male data were combined. Data are presented as mean ± SEM and analyzed using two-way (**A**) or one-way (**B**) ANOVA followed by Tukey’s multiple comparison test with * representing *p* < 0.05 in comparison with the control group (*n* = 12; 6 females/group and 6 males/group)

**Fig. 2:**
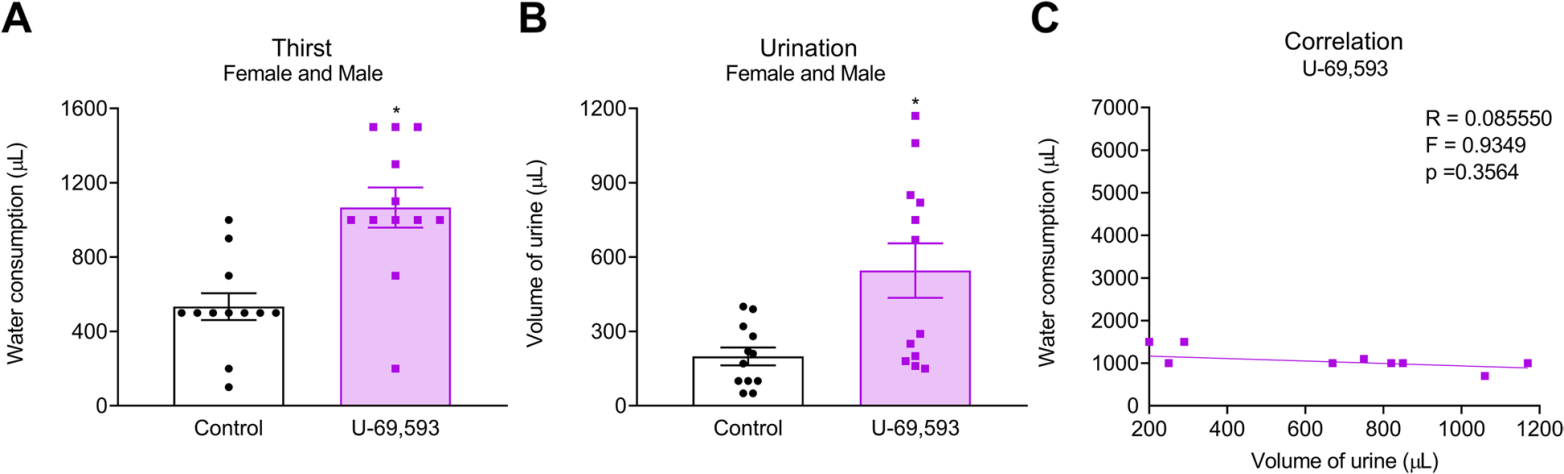
Systemic U-69,593 increased water consumption and urination in female and male mice. Mice were individually placed in metabolic chambers and received a single subcutaneous administration of U-69,593 at 30 mg/kg or vehicle-control. **A** Water consumption and **B** volume of urine, as outcome measurements of thirst and polyuria, respectively, were evaluated for 120 min after treatment. **C** Linear regression was performed to evaluate the correlation between water consumption and urination in U-69,593-treated mice. Female and male data were combined. Data are presented as mean ± SEM and analyzed using one-way ANOVA followed by Sidak’s multiple comparison test with * representing *p* < 0.05 compared to the control group (*n* = 12; 6 females/group and 6 males/group)

**Fig. 3:**
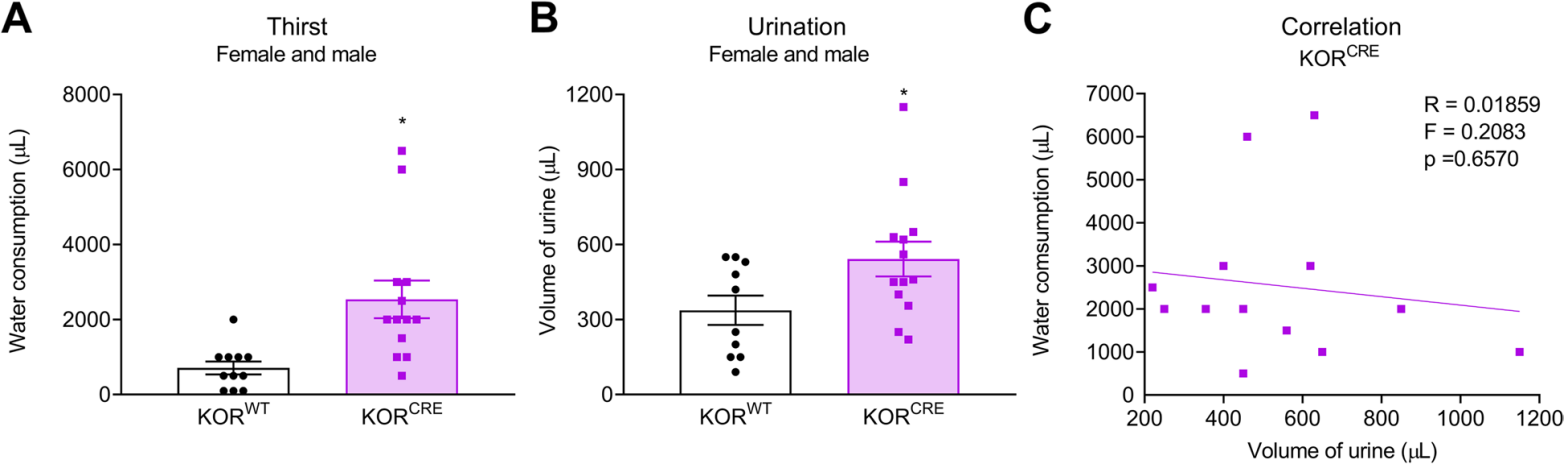
Chemogenetic manipulation of KOR^CRE^ neurons in the ARC increased water consumption and urination in both female and male mice. KOR^CRE^ or KOR^WT^ female and male mice were individually placed in metabolic chambers and received a single dose of CNO (DREADD specific agonist; 5 mg/kg, i.p.) four weeks after stereotaxic administration of AAV8-hSyn-DIO-hM4D(Gi)-mCherry virus (100 nL) into the right ARC. **A** Water consumption and **B** volume of urine, as outcome measurements of thirst and polyuria, respectively, were evaluated for 120 min after treatment. **C** Linear regression was performed to evaluate the correlation between water consumption and urination in KOR.^CRE^ mice. Female and male data were combined. Data are presented as mean ± SEM and analyzed using one-way ANOVA followed by Sidak’s multiple comparison test (**A**) and (**B**) with * representing *p* < 0.05 compared to the control group (*n* = 10 – 13; Panel A: 5 females/6 males WT and 6 females/7 males HET; Panels B and C: 5 females/5 males WT and 6 females/7 males HET)

**Fig. 4:**
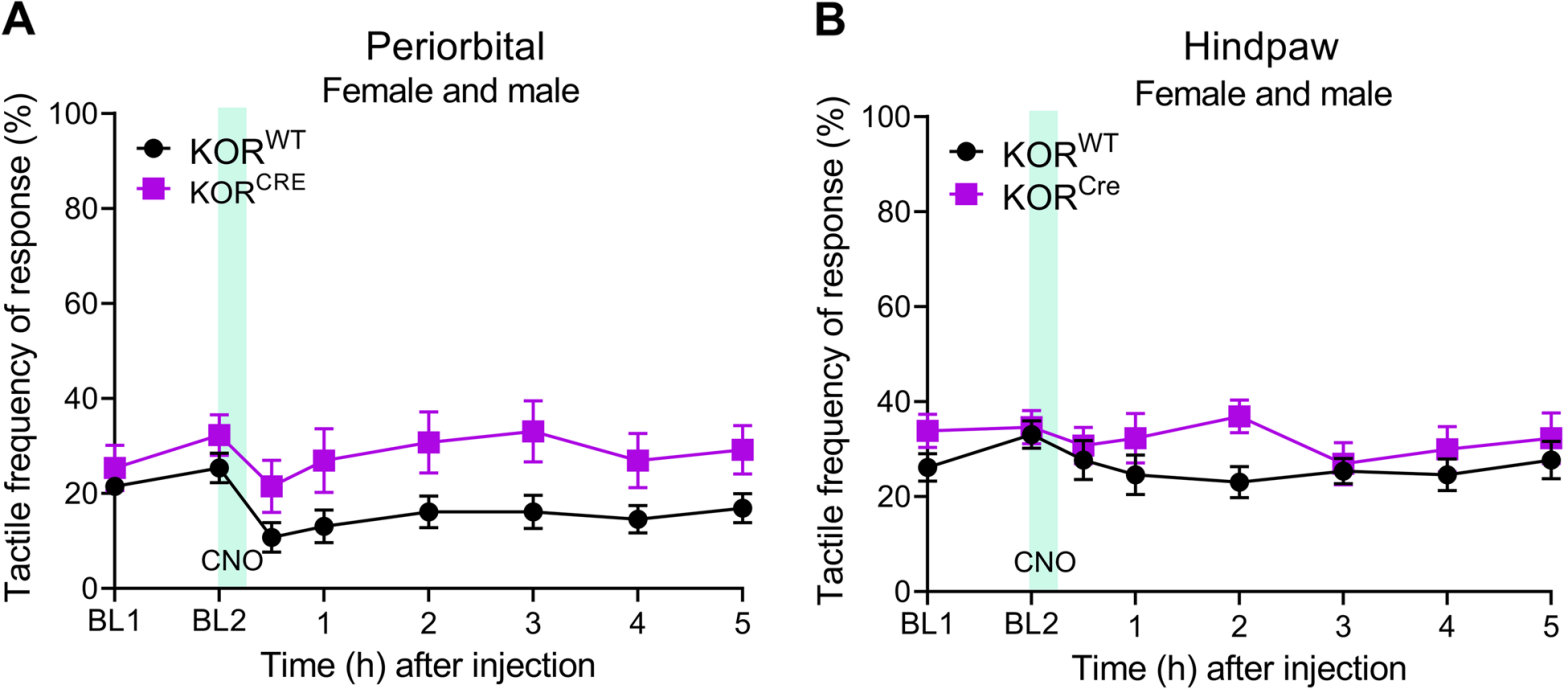
Chemogenetic manipulation of KOR^CRE^ neurons in the ARC did not modify periorbital and hindpaw tactile responses in female and male mice. Frequency of response to tactile stimulation in the **A** periorbital and **B** hindpaw region was performed before AAV virus injection (BL1), 4 weeks after the injection (BL2), and hourly up to 5 h after administration of CNO (5 mg/kg, i.p.) in KOR^CRE^ or KOR^WT^ mice. Data from female and male mice were combined and are presented as mean ± SEM. Statistical analysis was performed using two-way ANOVA followed by Sidak’s multiple comparison test (**A**) and (**B**) (*n* = 13; 7 females/6 males WT and 6 females/7 males HET)

The sample size was calculated using the GPower 3.1 software. Statistical analyses were calculated using GraphPad Prism 8 (GraphPad Software, La Jolla, CA, USA). D'Agostino-Pearson normality test was performed prior to the further statistical analysis. One or two-way analysis of variance (ANOVA) followed by Tukey’s post hoc test were used to analyze yawning, polyuria, and water consumption in rats. The Student’s t-test was used to analyze urination, and water consumption data in mice. Two-way analysis of variance (ANOVA) followed by the Sidak test was used for the analysis of the time course experiments for tactile sensory thresholds. Linear regression was performed to analyze the possible correlation between water consumption and volume of urine (urination). Data are presented as mean ± SEM, and “n” represents the number of animals analyzed. Statistical significance was set to an alpha level of 0.05. The numbers of animals used, *p* values, and F ratios are reported in Table 1. Due to the lack of statistical sex differences in the tests, male and female data were combined in Figs. 1, 2, 3 and 4.

**Table 1:** Summary of statistical analyses

Figure	Analysis	Interaction p	Interaction F	n
1A	Two-way ANOVA Tukey	*p* < 0.0001	F (6, 99) = 44.57	12 (6 female/group) (6 male/group)
1B	One-way ANOVA Tukey	*p* < 0.0001	F (2, 33) = 22.97	12 (6 female/group) (6 male/group)
2A	Student’s t-test	*p* = 0.0005	F = 2.223, 11, 11	12 (6 female/group) (6 male/group)
2B	Student’s t-test	*p* = 0.0066	F = 9.179, 11, 11	12 (6 female/group) (6 male/group)
2C	Linear regression	*P* = 0.3564	F = 0.9349	12 (6 female/group) (6 male/group)
3A	Student’s t-test	*p* = 0.0040	F = 10.06, 12, 10	11 – 13 (5 female/6 male WT) (6 female/7 male HET)
3B	Student’s t-test	p = 0.0427	F = 1.819, 12, 9	10 – 13 (5 female/5 male WT) (6 female/7 male HET)
3C	Linear regression	*p* = 0.6570	F = 0.2083	10 – 13 (5 female/5 male WT) (6 female/7 male HET)
4A	Two-way ANOVA Sidak	*p* = 0.7502	F (6, 144) = 0.5744	13 (7 female/6 male WT) (6 female/7 male HET)
4B	Two-way ANOVA Sidak	*p* = 0.3254	F (6, 144) = 1.17	13 (7 female/6 male WT) (6 female/7 male HET)
S1A	Student’s t-test	*p* = 0.9247	F = 3.979, 7, 7	8 (8 female/group)
S1B	Student’s t-test	*p* = 0.6243	F = 1.2, 7, 7	8 (8 female/group)

*p* values, interaction F ratios and n for statistical analyses used in Figs. 1, 2, 3 and 4 and supplementary figure S1

## Results

### Systemic KOR agonist increased urination but not yawning in both female and male rats

Subcutaneous administration of the potent D1 and D2 dopaminergic agonist, apomorphine at 0.05 mg/kg, induced a pronounced increase in yawning behavior in both female and male rats compared to the control group. In contrast, the systemic administration of the KOR agonist, U-69,593 at 0.56 mg/kg, failed to induce yawning behavior in rats (Fig. 1A). However, the injection of U-69,593 significantly increased urination (Fig. 1B) when compared to the control group. Control-treated rats did not demonstrate significant changes in the yawning behavior or urination throughout the experimental time course (Fig. 1A and B). The main purpose of this experiment was to determine if systemic administration of KOR agonist, U-69,593 would produce yawning behavior in rats and urination was measured as a secondary outcome and to confirm the engagement of KOR.

### Systemic U-69,593 increased water consumption and polyuria in female and male mice

Systemic administration of U-69,593 at 30 mg/kg (Fig. 2) increase both water consumption (Fig. 2A) and volume of urine (polyuria) (Fig. 2B) in female and male mice when compared to vehicle-treated mice. Linear regression revealed a lack of correlation between increased water consumption and volume of urine after U-69,593 administration (Fig. 2C). Water consumption (Supplemental Fig. 1A) and volume of urine (Supplemental Fig. 1B) was not altered after systemic administration of U-69,593 at a lower dose of 0.56 mg/kg in female mice.

### Chemogenetic activation of Gi-DREADD in KOR^CRE^ neurons in the ARC increased water consumption and urination in both female and male mice

Four weeks after the injection of AAV8-hSyn-DIO-hM4D(Gi)-mCherry virus into the right ARC, cre-dependent expression of hM4D(Gi)-mCherry (Gi-DREADD) (red) was confirmed in female (Supplemental Fig. 2A) and male (Supplemental Fig. 2B) KOR^CRE^ mice, but no expression was observed in KOR^WT^ littermates. Significantly increased water consumption (Fig. 3A) and volume of urine (Fig. 3B) was observed following a single systemic dose of CNO, a Gi-DREADD specific agonist, in KOR^CRE^/Gi-DREADD female and male mice in comparison to KOR^WT^ control groups. Linear regression revealed a lack of correlation between increased water consumption and volume of urine after CNO administration in KOR^CRE^/Gi-DREADD mice (Fig. 3C).

### Chemogenetic activation of hM4D(Gi) in ARC KOR^CRE^ neurons did not produce periorbital or hindpaw allodynia in female and male mice

Expression of Gi-DREADD in the right ARC did not modify baseline tactile frequency of response in the periorbital (Fig. 4A) and hindpaw (Fig. 4B) regions of KOR^CRE^/Gi-DREADD mice. Similarly, no changes were observed in KOR^WT^ mice that underwent the same stereotaxic surgery and virus injection but did not express Gi-DREADD. A single administration of CNO, 4 weeks after virus injection, did not modify periorbital (Fig. 4A) and hindpaw (Fig. 4B) frequency of response to tactile stimulation in KOR^CRE^/Gi-DREADD or KOR^WT^ female and male mice and there was no difference between the KOR^CRE and^ KOR^WT^ groups.

## Discussion

The hypothalamus is involved in the regulation of many aspects of the body homeostasis, adjusting the drive to eat, drink, sleep, and to control the body temperature according to necessity [32–34]. In addition, the hypothalamus can also be implicated in behaviors of expelling or retaining urine [33–36]. Clinical imaging studies have demonstrated hypothalamic activation prior to the migraine-related headache attack, consequently associating activation of this brain region with the premonitory phase [5, 14–16]. We can speculate that some premonitory symptoms might occur due to hypothalamic activation induced by an imbalance of the body homeostasis, which can be considered a stressor, for example, skipping meals, dysregulated sleep, not drinking enough water, events described as migraine triggers. Hypothalamic activation might ultimately engage the dynorphin/KOR stress system to produce some of the premonitory symptoms. Corroborating to this idea, increased levels of dynorphin in the hypothalamus have been observed in mice deprived of food and/or water, likely reflecting the generalized activation of stress circuits [37, 38]. The current study explored the possible link between KOR signaling in the hypothalamus in eliciting effects in rodents what could mimic, in part, symptoms observed during the premonitory phase of migraine including yawning, polyuria and increased thirst. Our data suggest that hypothalamic KOR activation promotes an increase in urination and thirst, but not yawning behaviors, without affecting tactile sensory thresholds, in female and male rodents.

Yawning is one of the symptoms reported by individuals with migraine during the premonitory phase [39]. Several hypothalamic nuclei have been implicated in the mediation of yawning, including the dorsomedial nucleus, ventromedial nucleus, and anterior hypothalamus, but mainly the paraventricular nucleus (PVN) [40–42]. Yawning behavior in rodents can be elicited through a multitude of mechanisms but include especially dopaminergic agonists. In this study, we used apomorphine, a dopaminergic D1/2 agonist to demonstrate yawning (i.e., as a positive control) [43]. Systemic administration of apomorphine produced robust and significant yawning behavior in rats, most likely through the activation of hypothalamic dopaminergic receptors as previously reported [43]. As hypothalamic KOR positive neurons are known to express dopamine [44–46], we hypothesized that KOR activation might promote increased yawning behavior. However, our data showed that systemic administration of U-69,593 failed to induce yawning behavior in female and male rats. Previous reports have shown that PVN administration of U-69,593 in rats did not affect yawning induced by systemic apomorphine or intracerebroventricular oxytocin, however, these authors did not evaluate the direct effect of hypothalamic administration of the KOR agonist on yawning [47]. Thus, the KOR may not play a significant role in the yawning behavior commonly observed in the migraine premonitory phase.

Polyuria and increased thirst are also commonly reported premonitory symptoms of migraine [1–3]. Our study revealed that systemic administration of U-69,593 increased urination in female and male rats and mice. It is important to point out that while different doses of U-69,593 were used in the mouse and rat experiments all doses revealed the metabolic effects resulting from KOR engagement. Systemic administration of KOR agonists has been widely reported to produce diuretic effects in many species [48–51] including humans [52–56]. Both central and peripheral mechanisms are associated with KOR-induced diuresis [57]. KOR agonists can act in the hypothalamus to produce diuresis [58–60]. Preclinical studies have suggested that KOR agonists induced diuresis by reducing the levels of vasopressin [61–64]. Peripheral mechanisms also contribute to modulation of sympathetic neural outflow to the kidneys from peripherally restricted KOR agonists which can also induce diuresis in rats [65–69]. Likewise, diuresis is a side-effect of CARA845 (i.e., Korsuva), a peripherally restricted KOR agonist that has recently been approved by the FDA for the treatment of itch [70]. Furthermore, we observed that systemic administration of U-69,593 increased water consumption in mice suggestive of increased thirst. Lee and colleagues have demonstrated that systemic administration of KOR agonists produced delayed increase in the water consumption of rats [71]. Thus, the increased urination and water consumption observed in our study following systemic KOR agonist are consistent with the hypothesis that KOR activation can promote polyuria and thirstiness associated with the premonitory phase of migraine but could result from central mechanisms, peripheral mechanisms, or both.

For this reason, we employed a chemogenetic strategy to determine if KOR expressing cells in the hypothalamus might be specifically responsible for polyuria and increased water consumption. We found that activation of a Gi-coupled DREADD (i.e., inhibition through Gi-coupled signaling) in KOR^CRE^ neurons in the ARC increased urination in both female and male mice. It should be noted that the Gi-DREADD expressing virus was injected unilaterally into the right ARC. Based on previous electrophysiological studies, the manipulation of neuronal activity on one side of this hypothalamic nucleus may affect the activity of the other side [72]. These findings are consistent with previous reports that demonstrate that hypothalamic administration of KOR agonists produced diuresis in rats [58, 60]. The hypothalamus is also known to receive inputs from lateral terminalis neurons, which sense and detect the necessity for fluid consumption [73, 74]. Mogenson and Stevenson demonstrated that electrical stimulation of the lateral hypothalamus (LHA) of rats increased water consumption [75], whereas lesion of the LHA caused dehydration [76, 77]. Herein, Gi-DREADD activation in KOR^CRE^ neurons in the mouse ARC with a single dose of CNO increased water consumption. Thus, our study revealed that activation of KOR in the ARC might play a significant role in diuresis and fluid intake homeostasis possibly associated with the premonitory migraine symptoms. It has previously been suggested that increased water consumption promoted by KOR agonists could be due to excessive urination [71]. However, we found no correlation between increased water consumption and increased urination after systemic administration of U-69,593 and after Gi-DREADD activation of KOR^CRE^ neurons, suggesting that these symptoms are unlikely to be causally related. Moreover, our data show that the increased urination and water consumption observed following ARC Gi-DREADD KOR^CRE^ activation did not alter periorbital and hindpaw tactile responses, suggesting that this mechanism might contribute to some of the premonitory symptoms in the absence of pain.

## Conclusions

The present study evaluated some proposed premonitory symptoms of migraine using multiple methods and approaches, as well as different rodent species and both sexes to provide increased rigor and confidence in conclusions. To our knowledge, this is the first preclinical study that attempts to unravel the mechanisms that may underlie the premonitory phase of migraine. Some limitations of our study should be noted. Imaging studies demonstrate activation of the hypothalamus as a whole in the premonitory phase, but here, we only studied the ARC nucleus. Other hypothalamic areas and mechanisms may play a significant role in these, or other premonitory symptoms. Sleep disruption is also observed in the premonitory phase of migraine. While the present study did not directly measure sleep after systemic administration of U-69,593 or Gi-DREADD KOR activation, recent work from our laboratory has demonstrated that KOR in the hypothalamic paraventricular nucleus promotes insomnia in mice [78]. Determining the role of KOR activation in different hypothalamic nuclei and in additional premonitory symptoms, including sleep, food craving, and mood change will require further investigation. Some reports have suggested differences in the dynorphin/KOR system in men and women in several brain regions, including the hypothalamus [79]. However, in the present study, we did not observe significant sex differences elicited by systemic administration of KOR agonist or hypothalamic KOR activation in increased urination or water consumption.

We previously reported that KOR antagonists might be considered for prevention of stress-related migraine [21, 24]. Consistent with this proposition, we now show that cell-specific manipulation of KOR-expressing neurons in the ARC of the hypothalamus of female and male mice produced polyuria and increased water consumption, but not yawning or pain responses, suggesting relevance to clinically observed premonitory symptoms of migraine. Understanding the neurobiology of the premonitory phase may allow for the development of treatments that may be given early in migraine attacks to prevent progression to a full-blown syndrome that includes the headache phase.

## Supplementary Information

**Additional file 1: Supplementary Fig. 1.** Systemic administration of the same dose of U-69,593 used in the rat study did not increase water consumption and urination in female mice. Mice were individually placed in metabolic chambers and received a single subcutaneous administration of U-69,593 at 0.56 mg/kg or vehicle-control. (**A**) Water consumption and (**B**) volume of urine, as outcome measurements of thirst and polyuria, respectively, were evaluated 120 minutes after treatment. Data are presented as mean ±SEM and analyzed using one-way ANOVA followed by Sidak’s multiple comparison test with * representing *p*<0.05 compared to the control group (*n* = 8 females/group).

**Additional file 2: Supplementary Fig. 2.** Representative images demonstrating the expression of Gi-DREADD-mCherry (red) in the ARC of female (**A**) and male (**B**) KOR^CRE^ heterozygous mice 4 weeks after stereotaxic administration of AAV8-hSyn-DIO-hM4D(Gi)-mCherry virus (100 nL) in the right ARC. Scale bars, 100 μm.

## Data Availability

All experimental data for this study are available from the corresponding author upon request.

## Abbreviations

ARC: Arcuate nucleus
CNO: Clozapine-N-oxide
DREADD: Designer Receptors Exclusively Activated by Designer Drugs
FDA: Food and drug administration
KOR: Kappa opioid receptor
LHA: Lateral hypothalamus
PAG: Periaqueductal gray
PVN: Paraventricular nucleus
s.c.: Subcutaneous
