# Supplementary figures and images for: Exploring the neurobiology of the premonitory phase of migraine preclinically – a role for hypothalamic kappa opioid receptors?
Source: J Headache Pain. 2022 Sep 30;23(1):126. doi: 10.1186/s10194-022-01497-7 (PMC9524131; doi:10.1186/s10194-022-01497-7)

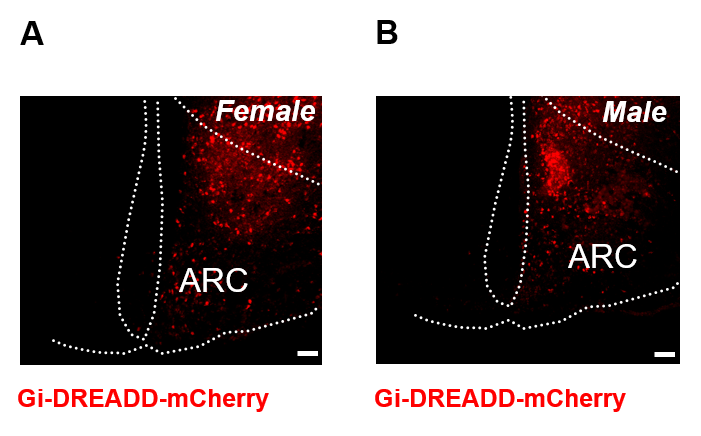

Supplement: Supplementary file 2 — Additional file 2: Supplementary Fig. 2. Representative images demonstrating the expression of Gi-DREADD-mCherry (red) in the ARC of female (A) and male (B) KORCRE heterozygous mice 4 weeks after stereotaxic administration of AAV8-hSyn-DIO-hM4D(Gi)-mCherry virus (100 nL) in the right ARC. Scale bars, 100 μm. [file 10194_2022_1497_MOESM2_ESM.tif]
